# Implementation of Instrumental Assessment to Assess Dysphagia in Older Adults Receiving Long-Term Care Services: A Scoping Review

**DOI:** 10.3390/geriatrics10020053

**Published:** 2025-04-03

**Authors:** Alvis Ki-Fung Kan, Elaine Kwong, Michael Siu-Wai Chan, Phoebe Tsz-Ching Shek

**Affiliations:** 1 Department of Rehabilitation Science, The Hong Kong Polytechnic University, Hong Kong, China; alvis.kan@connect.polyu.hk; 2 Department of Chinese and Bilingual Studies, The Hong Kong Polytechnic University, Hong Kong, China; 3 Research Institute for Smart Ageing, The Hong Kong Polytechnic University, Hong Kong, China; 4 Department of Biomedical Engineering, The Hong Kong Polytechnic University, Hong Kong, China

**Keywords:** dysphagia, instrumental assessment, implementation, community, long-term care

## Abstract

**Background/Objectives:** Dysphagia, a prevalent condition among older adults, poses significant health risks if not accurately assessed and managed. Instrumental assessments (IAs) like videofluoroscopic swallowing study (VFSS) and fiberoptic endoscopic evaluation of swallowing (FEES) allow detailed examinations of swallowing physiology but are underutilized in long-term care settings due to logistical challenges. This study aims to explore the current practice patterns, stakeholder perspectives, and barriers to and facilitators of IA implementation in these settings. **Methods:** A scoping review was conducted following the PRISMA-ScR guidelines, analyzing the literature from databases including CINAHL Complete, EMBASE, MEDLINE, and SCOPUS. A total of 1339 articles were identified. After the removal of 332 duplications, 1007 articles were screened, with four meeting the inclusion criteria for describing IA implementation or stakeholder perspectives in community-based long-term care settings for older adults. **Results:** This review identified significant underutilization of IA in long-term care settings, primarily due to logistical barriers and transportation issues. Stakeholders, particularly speech–language pathologists (SLPs), acknowledged the benefits of IA in improving dysphagia management but encountered challenges in accessing these assessments. Mobile FEES (mFEES) emerged as a promising solution, offering on-site assessments that could enhance the accuracy and timeliness of dysphagia care. **Conclusions:** While IA is crucial for effective dysphagia management in older adults, its implementation in long-term care settings is hindered by various barriers. mFEES presents a viable solution to improve IA accessibility and representativeness. Further research is warranted to develop context-specific implementation strategies and to explore the perspectives of all stakeholders involved in dysphagia care.

## 1. Introduction

Dysphagia refers to difficulty in the process of transiting food from the mouth to the stomach, which is commonly associated with neurological conditions such as stroke, Dementia and Parkinson’s disease [1]. Dysphagia is a common geriatric syndrome affecting 40–68% of adults [2]. A study conducted in Hong Kong revealed that 61% of older adults residing in long-term residential facilities and 41% of service users in day care centers presented with various forms and severities of dysphagia and required diet modification [3]. Accurate and timely assessment is essential to identify and prevent complications associated with dysphagia, including choking, aspiration pneumonia, malnutrition and death [4].

In Hong Kong, older adults aged 60 or above with proven needs are eligible to apply for long-term care services. Government-subsidized long-term care services include community services and residential services. Community services are non-residential in nature and comprise home care services (Integrated Home Care Services/Enhanced Home and Community Care Services) and Day Care Units for the Elderly. Residential services comprise care and attention homes and nursing homes [5]. Currently, older adults receiving long-term care services identified as requiring require instrumental assessment (IA) by their attending medical officer or speech–language pathologists (SLPs) are referred to outpatient clinics of the public (government-subsidized) or private hospitals (self-financed) and required to travel off-site for the assessment. In light of the limited availability of IA, clinical bedside swallowing evaluation (CBSE) is commonly performed as the primary assessment method by SLPs to examine the swallowing functions of older adults in long-term care settings in Hong Kong [6].

CBSE typically involves reviewing medical history, communication assessment, physical examination, and swallowing trials. Information such as general cognition, comprehension ability, and speech and language samples can be obtained in the communication assessment. In physical examination, SLPs examine the speech and swallowing structures, cranial nerves, voice, and laryngeal functions of older adults [6,7]. Swallowing trials are conducted subsequently to test for overt signs of aspiration with different fluid/food consistencies. CBSE is an efficient and affordable option for assessing dysphagia function in older adults since it requires little manpower and equipment. However, limitations such as limited physiology specificity, inconsistent results, high reliance on the clinician’s experience and variability in the components included in CBSE were reported in the literature [8].

On the contrary, IA, specifically videofluoroscopic swallowing study (VFSS) and fiberoptic endoscopic evaluation of swallowing (FEES), can provide more information about swallowing anatomy and physiology than CBSE alone because they allow direct visualization of swallowing and provide detailed information regarding the location and volume of food/fluid residue, the temporal aspect of the swallowing, and the detection of penetration or aspiration [2]. VFSS is a radiological imaging examination where radio-opaque food and liquid are swallowed. VFSS allows a dynamic evaluation of oropharyngeal swallowing function by providing real-time bolus flow and structural movement [9]. However, radiation exposure limits the frequency of VFSS examination. VFSS may not be appropriate for serial follow-ups due to radiation exposure [10]. In addition, VFSS has low portability since it must be conducted in radiation-proof facilities, which makes it inaccessible for some frail patients [11]. FEES involves the transnasal passing of a flexible endoscope with a light source and camera to observe the pharyngeal and laryngeal structures during swallowing [12]. The drawbacks of FEES include “white-out” due to swallowing materials covering the tip of the endoscope amid swallowing and discomfort caused by the insertion of an endoscopic tube [10,11].

In recent years, an emerging body of literature has proposed the utilization of ultrasound as an imaging technique in dysphagia assessment in adjunct to CBSE [10,13,14]. Ultrasound has provided an opportunity to assess swallowing at both the anatomical and kinematic levels. Ultrasound has been applied to assess tongue and posterior wall movement, hyoid displacement, vocal fold movement, and opening of the upper esophageal sphincter [14,15]. Ultrasound provides a more detailed depiction of oropharyngeal anatomy and suprahyoid musculatures than VFSS and FEES [14]. More importantly, ultrasound has several advantages over VFSS and FEES since it is non-invasive, involves no radiation, and causes negligible disturbance to the swallowing process [10,14]. High-resolution manometry (HRM) involves the placement of a pressure-sensing catheter through the pharynx and esophagus. The pressure-sensing catheter collects information regarding pharyngeal pressure during swallowing [16]. Although HRM does not provide information that is obtainable in VFSS or FEES, such as pharyngeal mobility, pharyngeal residue, and aspiration of the bolus, it measures pharyngeal pressure during swallowing, which provides information regarding the biomechanics of the swallowing physiology [16,17]. Biomechanics information of the swallowing physiology can be combined with the results of other instrumental assessments (e.g., VFSS and FEES) to infer the underlying etiologies of pharyngeal dysphagia [17]. Studies have reported the use of HRM alone or in combination with other imaging modalities such as VFSS or FEES to aid the evaluation of pharyngeal dysphagia [17,18].

IA provides more information regarding swallowing safety and efficiency than CBSE alone since it allows a more comprehensive examination of the swallowing physiology to aid in the identification of swallowing impairment. In addition, it facilitates treatment planning that directly targets the impairment [19]. Despite its critical role in assessing swallowing safety and efficiency, IA has seldom been performed in community and long-term care settings in Hong Kong. Japan, on the other hand, allows physicians and dentists to perform FEES at patients’ bedside or during a home visit [20]. The under-utilization of IA may fail to cater to the healthcare needs of older adults with dysphagia receiving long-term care services.

The literature related to the utilization of IA in older adults in long-term care settings is limited. A scoping review by Birchall et al. [8] summarized the use of IA in adults in residential aged care homes (RACHs). The study reviewed sources such as peer-reviewed journals, gray literature, and websites of IA providers to identify the application of VFSS and FEES in RACHs. The authors reported that RACHs in Japan and the United States had made a recent effort to offer mobile FEES (mFEES) services, which allowed RACH residents to receive on-site FEES [8]. However, the existing literature still suggests the under-utilization of IA in RACHs [2]. Possible reasons contributing to the low utilization rate of IA for RACH residents, such as limited RACH staff/client knowledge about IA, gaps in clinical governance, a lack of on-site IA, and transportation issues, were reported [8]. Although the review by Birchall et al. [8] provided insight into the current practices and challenges in the utilization of IA in older adults, it had some shortcomings that require further investigation. Firstly, the review only focused on a specific long-term care setting (i.e., RACHs). The review did not report the application of IA in other long-term care settings apart from RACHs, for instance, in community-dwelling older adults receiving long-term care services. Secondly, the types of IA utilization were limited to VFSS and FEES. The review did not include studies that examined the use of other forms of IA, such as ultrasound and HRM. To provide a more comprehensive illustration of IA application in long-term care settings, this scoping review extends the context of long-term care settings to cover a broader range of settings such as day care centers and the community. Moreover, this scoping review provides a broader definition of IA to include ultrasound and HRM in addition to VFSS and FEES to gain a better understating of the overall application of IA.

In addition to this, the review by Birchall et al. [8] focused on exploring the use and evidence of IA in RACHs, and did not specifically study the perspectives of different stakeholders involved in the implementation of IA. To inform a practical and feasible implementation strategy of IA in long-term care settings, it is imperative to conduct a scoping review to speculate the current practice of IA provision to older adults in different long-term care settings worldwide as well as establish a thorough understanding of the perspectives of different stakeholders involved in dysphagia management regarding the implementation of IA. This review aims to identify possible service delivery models for the implementation of IA for older adults in long-term care settings and highlight existing barriers and enablers to facilitate the development of feasible and practical implementation strategies. Specifically, the following research questions will be addressed:

(1) What are the current practice patterns of IA provision to older adults in long-term care settings?

(2) How do different stakeholders (e.g., clients, SLPs, caregivers, healthcare managers, etc.) think about the utilization of IA in long-term care settings?

(3) What are the existing barriers to and enablers of implementing IA in older adults in long-term care settings?

The population, concept, and contextual framework were recommended as a guide to establish clear objectives and eligibility criteria for a scoping review [21]. In the present review, the populations targeted were older adults aged 60 or above receiving long-term care services and stakeholders involved in dysphagia care in long-term care settings.

The concepts of interest included the utilization or implementation of IA (e.g., VFSS, FEES, ultrasound, or HRM) to detect dysphagia in older adults receiving long-term care services or studies that reported stakeholders’ perspectives on the utilization or implementation of IA in long-term care settings. The context of this review is long-term care settings, in which long-term care settings are defined as day care center services, home care services, or residential care services.

## 2. Methods

This scoping review adopts a systematic approach suggested by the published guideline of the Preferred Reporting Items for Systematic Reviews and Meta-Analyses Extension for Scoping Reviews (PRISMA-ScR) [22].

### 2.1. Protocol and Registration

The protocol of this study was developed according to the PRISMA-ScR guidelines [22] and registered with the Open Science Framework (https://osf.io/r7y2w) (accessed on 28 March 2025).

### 2.2. Eligibility Criteria

To be included in this review, the identified articles had to describe or evaluate the implementation or practice pattern of IA in a long-term care setting for older adults. Additionally, quantitative or qualitative research that investigated the perspectives of stakeholders regarding the implementation of IA in long-term care settings was also included. Studies that were not written in English were excluded. Studies without empirical quantitative or qualitative data regarding the implementation of IA, editorials, textbooks, systematic reviews, literature reviews, and study protocols were excluded. This review focuses on the implementation of IA in long-term care settings; thus, studies conducted in hospitals and acute settings were also excluded.

### 2.3. Information Sources

Databases including CINAHL Complete, EMBASE, MEDLINE, and SCOPUS were searched (March 2024). No date limits were applied to capture as many relevant studies as possible. The search strategy was drafted by the author and further refined by team discussion with two independent researchers (MC and PS). A consultation with a librarian was also conducted to review the search strategy. The author and the researchers were qualified SLPs with at least three years of clinical and research experience in older adults with dysphagia. Citations were imported to Endnote 21, and duplications were removed.

### 2.4. Search

The search terms were divided into five elements, namely “instrumental assessment”, “dysphagia”, “older adults”, “implementation”, “stakeholders’ perspective” and “long-term care setting”. Alternatives were derived from the five elements. The five elements were combined with the Boolean operator “AND”. The search terms used for the database search are summarized in Table 1.

### 2.5. Selection of Source of Evidence

The screening of identified articles adhered to the recommendations proposed by the published guidelines for conducting a scoping review [23]. The author and the two independent researchers conducted a calibration exercise by screening 10% of the identified articles independently, aiming for a 90% level of agreement. After calibration was achieved, the author and the reviewers screened the remaining articles independently. Verification of the results was carried out to ensure a level of agreement of 90% or above was achieved. Disagreements on publication selection were resolved among the author and reviewers by consensus. Discussion with a third party would be arranged if needed. Articles that met the inclusion criteria were read in full to determine the final inclusion of articles for this review.

### 2.6. Data Charting Process

A charting form for data extraction was developed by the author and reviewed by the two reviewers. The data charting was performed by the author using the charting form designed for this study. The charting form captured the relevant information on study characteristics and detailed information on all domains related to the research questions. The results were verified by the reviewers independently. Disagreements were resolved through discussion until consensus was reached among the author and reviewers.

### 2.7. Data Items

Information related to the study characteristics and research questions were extracted from the included studies. The data extracted included the author(s), title, year of publication, country, population/sample size, study design, long-term care setting addressed, type of instrumental assessment addressed, current practice patterns of IA, stakeholders’ perceptions of IA, barriers, and enablers to IA provision in long-term care settings.

### 2.8. Synthesis of Results

The results of the included studies were organized according to the research questions. The results were reported as a narrative summary for each research question of this scoping review.

## 3. Results

**Figure 1:**
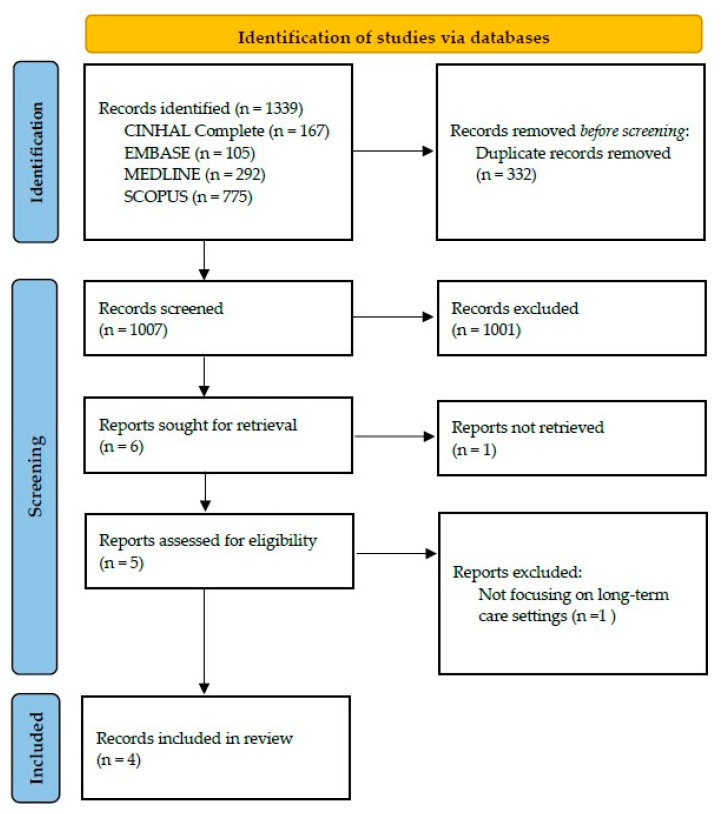
PRISMA flow diagram.

A total of 1339 articles were identified from the databases (CINHAL Complete: 167; EMBASE: 105; MEDLINE: 292; SCOPUS: 775). After the removal of duplicates, 1007 articles remained for screening. After title and abstract screening, 1001 articles were excluded, which left six articles to be assessed for eligibility. One article was excluded due to the unavailability of the full text. The remaining five articles were read in full, and one article was excluded because it focused on FEES in a specific patient group (geriatric schizophrenic patients) instead of long-term care settings. A total of four articles were included in this review (see Figure 1 for the PRISMA flow diagram).

### 3.1. Characteristics of the Included Studies

The characteristics of the included studies are summarized in Table 2. The studies included in this review originated from two different countries, namely Australia (*n* = 3) and Japan (*n* = 1). The long-term care settings addressed in these studies were mainly residential, including RACHs [2,24] and nursing homes [25]. One study focused on dysphagia care in the community [26]. All included studies addressed the use of FEES, and three of them addressed both FEES and VFSS [2,24,26]. Only one study implemented IA directly in older adults in long-term care settings [25]. The study implemented FEES in nursing home residents who screened positive for dysphagia to determine the characteristics of dysphagia. The other three studies investigated the practice patterns and SLPs’ perceptions of the implementation of IA in long-term care settings, which did not involve the direct application of IA in older adults. In terms of study methods, one of these three studies employed the modified Delphi consensus method to explore the role and use of IA in RACHs from the perspective of SLPs [2]. One study retrospectively reviewed the medical records of RACH residents to investigate the relationship between the indication of IA and the practice patterns of SLPs [24]. One study explored SLPs’ practice patterns for IA in a community setting by conducting online surveys [26].

### 3.2. Current Practice Patterns of IA (Research Question #1)

Two studies described the provision pattern of IA at RACHs in Australia [2,24]. Residents who were identified as requiring VFSS or FEES had to travel off-site to an outpatient clinic (hospital-based) to receive the service. Another study focused on community dysphagia care services in Australia that reported limited access to IA for some community-based SLPs [26]. Only a small proportion of SLPs had direct access to VFSS or FEES within their workplace, while the rest of them relied on indirect access to IA through other local services (see Table 3).

These studies highlighted the essential role of IA in providing more accurate assessment results and developing higher-quality treatment plans than CBSE alone [2,24,26]. Despite the essential role of IA in dysphagia assessment and management, the under-utilization of IA in long-term care settings was reported in two studies [2,24]. Two studies reported concerns about assessment and treatment planning validity as well as potential harm (e.g., malnutrition, dehydration, decreased quality of life) to older adults with unnecessary and over-conservative diet modification in the absence of IA [25,26]. The study by Birchall et al. [24] reviewed 115 medical records of RACH residents; 33 residents whose records were indicative of a need for IA were not referred for IA. Out of those 33 cases, 20 cases presented an unclear nature of dysphagia that might warrant IA, and 13 cases might benefit from IA for dysphagia management (e.g., trial of compensatory strategies or biofeedback).

### 3.3. Stakeholders’ Perspectives on the Implementation of IA in Long-Term Care Settings (Research Question #2)

Stakeholders’ perspectives on IA implementation in long-term care settings are summarized in Table 4. One study conducted a modified Delphi survey to investigate SLPs’ perception of the implementation of IA in residential aged care homes in Australia [2]. The study reported that 89.7% (52/58) of respondents reached a consensus that clients who were diagnosed with dysphagia by SLPs should have access to IA. SLP participants agreed on three major benefits of IA provision for residential aged care home residents with dysphagia, including improvement in swallowing-related quality of life through informed dysphagia management (87.9%, 51/58), reduced healthcare costs due to the minimization of complications associated with dysphagia (79.3%, 46/58), and reduced preventable emergency department admission (79.3%, 46/58). Specifically, 81.1% (47/58) of participants acknowledged the benefit of FEES for family, client, and staff education in dysphagia care. However, a discrepancy was noted between the perceived importance and the actual provision pattern of IA. The study revealed that 74.2% (43/58) of respondents agreed that IA was underutilized in RACHs [2] (See Table 4).

### 3.4. Barriers to and Enablers of the Implementation of IA in Long-Term Care Settings (Research Question #3)

Table 5 summarizes the barriers to the implementation of IA in long-term care settings. Plenty of barriers were identified from the included studies. Access barriers and transportation issues were the main concerns in providing IA for older adults in RACHs. A study reported that SLPs in community settings lacked direct access to IA [26]. Most community-based services depended on referrals to other services to gain access to IA. A similar situation was observed in RACHs. Another study reported that RACH residents must travel off-site for IA due to access barriers to IA at RACHs, which induced significant transportation costs that could be prohibitive for some residents [24]. A third study reported that 71.1% (32/45) of SLP respondents agreed that the low availability of IA services at RACHs prevented residents from receiving timely IA services [2]. The study also reported that 94.5% (52/55) of SLP respondents indicated that the burden of traveling/unavailability of trained support staff to accompany residents for IA were barriers related to the under-utilization of IA in RACHs. Furthermore, the study reported that 88.9% (40/45) of SLP respondents believed that the absence of on-site IA services was a barrier to representative IA results.

Two studies reported health status as a barrier to providing IA to older adults in long-term care settings. A study reported that 73.3% (33/45) of SLP respondents agreed that the health status of older adults could be a barrier to timely and representative IA [2]. Another suggested that performing IA on older adults receiving advanced palliative care or with severe cognitive impairment could increase the risk of endoscope intolerance during the procedure due to a decreased level of compliance and elevated agitation level [25].

Limited staff education and knowledge of IA were also reported to be barriers to implementing IA in long-term care settings. A study suggested that SLPs might not recognize the indication for IA [24]. Another study reported that 86.7% (39/45) of SLP respondents agreed that staff and client knowledge of IA could prevent residents from receiving timely IA [2]. A study also pointed out that in Australia, SLPs must receive advanced training to conduct and interpret FEES, which is not usually feasible as most healthcare settings are not structured in a multidisciplinary way [26].

Enablers for developing IA services in long-term care settings were identified in two studies (see Table 6). One study reported that 89.5% (34/38) of SLP respondents believed that enhancing communication between the SLPs making IA referrals and the IA provider was an enabling factor to facilitate IA implementation in RACHs [2]. Specifically, a consistent point of contact should be established between the SLPs making IA referrals and the IA providers to discuss patient care. In addition, an effective IA implementation process including timely IA referrals and appropriate prioritization of clients could be achieved through strong collaboration between the SLPs making IA referrals and the IA providers. Another study suggested two facilitating factors for SLPs to initiate timely and appropriate IA referrals. Firstly, the development of evidence-based IA referral guidelines could assist SLPs in identifying indications of IA specific to RACH settings. Secondly, FEES education and training could also enhance the competence of SLPs to initiate timely and appropriate IA referrals [24].

## 4. Discussion

The objectives of this scoping review are to summarize the current practice patterns of IA in various long-term care settings, understand different stakeholders’ perspectives on the utilization of IA in long-term care settings, and identify the existing barriers to and enablers of implementing IA in older adults in long-term care settings. This review included long-term care settings beyond RACH settings and considered other forms of IA (i.e., ultrasound and HRM) apart from VFSS and FEES that were not addressed in previous studies. To the best of the authors’ knowledge, this is the first scoping review attempting to understand stakeholders’ perceptions and the practice patterns of IA across different long-term care settings. The results from the included studies indicated the significant role of IA in dysphagia care, as it provides important information on swallowing safety and efficiency, which are, in turn, essential for accurate diagnoses and effective treatment planning. However, the availability of IA to older adults in long-term care settings was limited. The studies identified major barriers attributed to the discrepancy between the need for and actual provision of IA for older adults in long-term care settings, including the lack of on-site services and the costs and time associated with off-site assessments. The underutilization of IA may negatively impact older adults’ quality of life due to the over-conservative management of dysphagia [25,26]. A study reported that as many as 91.1% (41/45) of respondents agreed that the absence of timely IA services led to over-conservative dysphagia management in RACHs [2]. This may also explain the high prevalence of liquid and food texture modification in RACHs in Hong Kong. A study reported that as many as 71.1% and 67.2% of RACH residents in Hong Kong required liquid and food texture modification, respectively. Texture-modified foods and liquids compromise the nutritional value and appearance of the diet, increasing the risks of malnutrition and reducing quality of life [28]. Therefore, IA in long-term care settings is useful to assist SLPs in making appropriate dysphagia management decisions.

mFEES may offer a promising solution to the underutilization of IA in long-term care settings. First, the cost of VFSS can be significantly higher than that of FEES due to the involvement of radiation equipment and operation specialists. On-site mobile VFSS services are also impossible due to the requirement of a radiation-proof facility and the low portability of radiation equipment. On the other hand, FEES is a portable, affordable, and low-risk option that provides crucial information on swallowing safety and efficiency without radiation exposure [29]. The results of this review suggested that mFEES is a possible service delivery mode to resolve time/cost concerns regarding transportation and improve the timeliness of IA. In addition, a study reported that 92.3% (48/52) of SLP respondents were skeptical about the representativeness of off-site IA because it might not reflect the daily mealtime performance of older adults in their residential settings [2]. The study also reported that 73.7% (28/38) of respondents were concerned about the representativeness of off-site IA results due to the absence of a familiar mealtime assistant, which might influence older adults’ performance in IA [2]. One advantage of mFEES is that older adults can receive assessments in familiar places, which reduces anxiety levels, especially for cognitively impaired individuals. Daily foods and beverages can be used in the assessment with the mFEES service to enhance the validity of the IA results. The study also showed that SLPs held a positive attitude towards mFEES [2]. The results indicated that 86.3% (50/58) of participants believed there were benefits to offering FEES in RACHs with a mobile on-site service model. Of these participants, 100% (50/50) of them agreed that it could eliminate the need for travel, 82% (41/50) of them agreed that it could provide more timely assessment, and 94% (47/50) of them agreed that the on-site FEES service allowed a familiar environment for assessment. Additionally, 81.1% (47/58) of respondents believed that the mFEES service could improve advanced care planning and enable more informed quality-of-life and medical decisions regarding swallowing management. However, the study also pointed out that 57.9% (22/38) of SLP respondents expressed concerns about the implementation of mFEES. Concerns were mainly related to resources (equipment and cost of operation); instrumentation sterilization, storage, and maintenance; and quality of care (SLPs’ knowledge on FEES and client access to multidisciplinary team support). On the other hand, the study reported that 77.6% (45/58) of SLP respondents believed that mFEES could be provided safely in RACHs with optimization of the physical environment, equipment, staff allocation, established procedures, and emergency plans [2]. It is noteworthy that staff education and knowledge of IA as well as residents’ health status were reported to be barriers to IA utilization in RACHs. These concerns must be addressed in the implementation of mFEES services. For instance, it is vital to educate SLPs and staff in long-term care settings on the function and indications of FEES. Furthermore, as older adults receiving long-term care services may exhibit agitation due to impaired cognition, or frailty due to advanced medical conditions, that may impede the validity of FEES results, it is important to establish evidence-based guidelines to determine appropriate candidacy for FEES.

The literature on this topic is scarce, indicating a huge research gap that can be addressed by future studies. Although the review by Birchall et al. [8] presented the use of mFEES in the United States and Japan, no retrievable published articles were found in the database searches that described the implementation and service delivery model of mFEES. In addition, the existing literature mainly focused on RACHs, with only one study conducted focusing on community settings. Since long-term care services encompass community and residential services, the existing literature might not be representative enough to illustrate the implementation of mFEES as an IA exemplar in long-term care settings. With the Hong Kong government’s advocacy of “ageing in place as core, institution as back-up”, community-based long-term care services play a vital role in supporting the increasing geriatric population residing in the community. An implementation study of IA in various long-term care settings (e.g., daycare centers, care and attention homes, nursing homes) is warranted to facilitate the development of implementation strategies specific to different long-term care settings. The present review summarized concerns related to mFEES implementation that ought to be addressed in future implementation studies, including costs, equipment cleaning and maintenance, and SLPs’ knowledge of FEES (especially regarding the benefits/limitations of FEES and indications for FEES referral). An implementation study that adopts a comprehensive implementation framework is required to address different inner and outer factors and integrate stakeholders’ perspectives across different long-term care settings. In addition, the implementation of other imaging techniques in long-term care settings, such as ultrasound, was not reported in the existing literature. Ultrasound was reported to provide valuable information on swallowing kinematics and oropharyngeal anatomy [14,15], which can be considered in future implementation studies in addition to FEES.

This review intended to include studies that investigated different stakeholders’ perceptions regarding the implementation of IA in long-term care settings. Nevertheless, the existing literature only reported opinions from SLPs. No study interviewed other stakeholders from dysphagia care teams. Although an SLP is generally considered a key member of the dysphagia care team [30], it is important to acknowledge the perspectives of other professionals in the multidisciplinary team and service users to gain a full understanding of the challenges and desired service delivery modes from different perspectives. Future implementation studies can include qualitative interviews with different stakeholders such as SLPs, nurses, healthcare managers, clients, and caregivers.

This scoping review provided a broad picture of the current developmental progress in the implementation of IA in long-term care settings. A huge discrepancy exists between the need for IA and the actual IA services provided in long-term care settings. There is also a significant lack of both qualitative and quantitative empirical evidence of IA implementation in long-term care settings. This review sheds light on possible future implementation studies of IA that involve various long-term care settings and perspectives from different stakeholders in dysphagia teams to reconcile the identified barriers and inform feasible implementation strategies of IA in different long-term care settings.

## 5. Conclusions

The use of IA to facilitate more accurate swallowing assessment and intervention planning has been well documented in the previous literature. The results of this review suggested that SLPs in long-term care settings held positive attitudes toward the use of IA in long-term care settings. The mFEES service delivery model may be a promising solution to the underutilization of IA in long-term care settings due to the well-established evidence of its efficacy in assessing swallowing safety and efficiency, high portability, and relatively low establishment and operation costs. A significant lack of existing evidence was noted to inform the implementation of mFEES and other types of IA in long-term care settings. A comprehensive implementation study of mFEES or its combination with other IA modalities (e.g., ultrasound) in different long-term care settings is warranted to develop feasible context-specific implementation strategies.

## Figures and Tables

**Table 1:** Search strategy.

Element	Alternatives
“Instrumental assessment”	“instrument” “instrumental” “videofluoroscopic swallowing study” “VFSS” “modified barium swallow” “MBS” “fiberoptic endoscopic evaluation of swallow” “FEES” “FEEST” “endoscopy” “endoscopic” “laryngoscopy” “ultrasound” “ultrasonography” “ultrasonographic” “sonography” “sonographic” “manometry” “manometric” “HRM”
“Dysphagia”	“dysphagia” “swallow” “deglutition” “eating” “feeding”
“Older adults”	“elderly” “adult” “geriatric” “older person”
“Implementation”/“Stakeholders’ perspective”	“implementation” “utilization” “practice” “perspective” “barrier” “challenge” “disabling” “facilitator” “enabling” “survey” “interview” “perception” “opinion” “comment” “view”
“Long-term care setting”	“community” “home” “primary care” “long term care” “residential care” “nursing home” “assisted living” “infirmary”
Boolean operators	“instrument” OR “instrumental” OR “videofluoroscopic swallowing study” OR “VFSS” OR “modified barium swallow” OR “MBS” OR “fiberoptic endoscopic evaluation of swallow” OR “FEES” OR “FEEST” OR “endoscopy” OR “endoscopic” OR “laryngoscopy” OR “ultrasound” OR “ultrasonography” OR “ultrasonographic” OR “sonography” OR “sonographic” OR “manometry” OR “manometric” OR “HRM”
	“dysphagia” OR “swallow” OR “deglutition” OR “eating” OR “feeding”
	“elderly” OR “adult” OR “geriatric” OR “older person”
	“implementation” OR “utilization” OR “practice” OR “perspective” OR “barrier” OR “challenge” OR “disabling” OR “facilitator” OR “enabling” OR “survey” OR “interview” OR “perception” OR “opinion” OR “comment” OR “view”
	“community” OR “home” OR “primary care” OR “long term care” OR “residential care” OR “nursing home” OR “assisted living” OR “infirmary”

**Table 2:** Characteristics of included studies.

Author(s) and Year (Country)	Study Aim	Study Design	Sample Size	Settings	Type of IA Addressed
Birchall et al. [2] (Australia)	To explore the role and use of IA in RACHs from SLPs’ perspective	Three-round electronic modified Delphi consensus study	58 SLPs familiar with RACHs or IA (VFSS or FEES)	RACHs	VFSS and FEES
Birchall et al. [24] (Australia)	To describe practice patterns of IA in RACHs	Retrospective study of medical records	323 medical files of RACH residents were reviewed	RACHs	VFSS and FEES
Howells et al. [26] (Australia)	To investigate community-based SLPs’ practice patterns in dysphagia care	Online survey	144 SLPs working with community-based clients with dysphagia	Community-based	VFSS and FEES
Imaizumi et al. [25] (Japan)	To investigate the prevalence and characteristics of swallowing impairment in older adults residing at care facilities	Questionnaire screening for dysphagia; FEES for those who screened positive for dysphagia in the questionnaires	413 nursing home residents	Nursing homes	FEES

**Table 3:** Current practice patterns of IA.

Author(s) and Year	Current Practice Patterns of IA
Birchall et al. [2]	- RACH residents must travel off-site to an outpatient clinic for IA services - Assessment is subject to multiple conditions, including transportation arrangement, arrangements for escorting staff, residents’ cognitive capacity to follow instructions, and financial considerations
Birchall et al. [24]	- Residents identified as requiring an IA by a doctor or SLP must be referred to an outpatient (usually hospital-based) IA clinic and travel off-site for assessment - No residents were referred for IA. Among 115 residents referred to SLP for swallowing assessment, 33 cases were indicated for IA according to indicators described by Langmore and Aviv [27]. Out of the 33 cases, 20 cases demonstrated unanswered diagnostic questions/an unclear nature of dysphagia that might indicate IA, and 13 cases demonstrate that they might benefit from IA to help guide dysphagia management (e.g., biofeedback or trial of compensatory strategy)
Howells et al. [26]	- Only a small proportion of SLPs had direct access to instrumental assessments within their workplace, and over half could gain indirect access to IA through the other local services

**Table 4:** Stakeholders’ perceptions of IA.

Author(s) and Year	Stakeholders’ Perceptions of IA
Birchall et al. [2]	On access to IA RACH residents with dysphagia should have access to IA (89.7%, 52/58) On benefits of IA IA improves swallowing-related quality of life through informed dysphagia management (87.9%, 51/58) IA minimizes physical and psychosocial complications of dysphagia and reduces healthcare expenditure (79.3%, 46/58) IA minimizes emergency department admissions (79.3%, 46/58) FEES recordings are useful in educating the patients, families, and RACH staff involved in dysphagia management (81.1%, 47/58) On current IA service IA is underutilized in RACHs (74.2%, 43/58) SLPs are reluctant to suggest FEES due to access barriers (72.4%, 42/58) On desired IA characteristics Meals during IA should resemble the patient’s daily meal environment to reflect usual swallowing function (88%, 51/58) IA should be arranged in a timely manner (84.5%, 49/58), ideally within 4–14 days from referral (84.5%, 37/44) On mFEES service mFEES has an advantage over off-site FEES (86.3%, 50/58) On advantages of mFEES Eliminates transportation time/cost concerns and challenges (100%, 50/50) Allows familiar and natural environment for IA assessment (94%, 47/50) Provides better education to RACH staff and residents’ families/caregivers (92%, 46/50) Customizes IA according to the client’s needs (84%, 42/50) Improves timeliness of assessment (82%, 41/50) Informs dysphagia management plans (81.1%, 47/58) Informs quality of life and medical care decisions (81.1%, 47/58) Facilitates the design of effective and efficient dysphagia therapy programs (77.6%, 45/58)

**Table 5:** Barriers to IA provision in long-term care settings.

Author(s) and Year	Barriers to IA Provision in Long-Term Care Settings
Birchall et al. [2]	Barriers to timely IA: Transportation (94.5%, 52/55); Knowledge (86.7%, 39/45); Governance (77.3%, 34/44); Financial management (75.6%, 34/45); Health status (73.3%, 33/45); Unavailability of IA services (71.1%, 32/45). Barriers to representative IA: Unfamiliar environment (92.3%, 48/52); Absence of on-site IA services (88.9%, 40/45); Unfamiliar mealtime assistants (73.7%, 28/38); Adults receiving end-stage palliative care or presenting with severe cognitive challenges (e.g., agitation) that increased risks of FEES-associated complications were not offered IA (73.3%, 33/45). Consequences of lack of IA A lack of timely access to instrumental swallowing assessment can result in overly conservative dysphagia management (91.1%, 41/45). Barriers to mFEES Resources (e.g., instrumentation, cost to consumer and health service) (71.1%, 27/38); Instrumentation cleaning, storage, transportation, and maintenance (78.3%, 18/23); Quality of care (e.g., skills of SLPs, timely medical input, quality of FEES service) (76.3%, 29/38).
Howells et al. [26]	Access to IA was a noted issue. Most community-based services relied on referral to other services to gain access to IA. Australian SLPs must receive advanced training to conduct and interpret FEES, which can only occur in multidisciplinary healthcare settings.
Imaizumi et al. [25]	Due to the need for patient compliance and the required expertise, it is not feasible to perform IA on every individual with suspected dysphagia.

**Table 6:** Facilitators of IA provision in long-term care settings.

Author(s) and Year	Facilitators of IA Provision in Long-Term Care Settings
Birchall et al. [2]	- mFEES can be performed safely in a RACH with appropriate processes, procedures, ancillary staff, equipment, and emergency management plans (77.6%, 45/58) - Additional training in FEES benefits and limitations (100%, 38/38) - Additional training in FEES referral criteria (94.7%, 36/38) - Enhanced communication with SLPs who conduct FEES (89.5%, 34/38)
Birchall et al. [24]	- Establish clear evidence-based referral guidelines for IA to facilitate identification of IA need - FEES education and training to support SLPs in initiating timely and appropriate IA referrals

## Data Availability

The original contributions presented in this study are included in the article. Further inquiries can be directed to the corresponding author.

## Abbreviations

The following abbreviations are used in this manuscript:

IA	Instrumental assessment
CBSE	Clinical bedside swallowing evaluation
VFSS	Videofluoroscopic swallowing study
FEES	Fiberoptic endoscopic evaluation of swallowing
HRM	High-resolution manometry
RACH	Residential aged care home
SLP	Speech–language pathologist
mFEES	Mobile fiberoptic endoscopic evaluation of swallowing
